# Perceptions towards management of acute malnutrition by community health volunteers in northern Kenya

**DOI:** 10.1371/journal.pgph.0002564

**Published:** 2024-05-16

**Authors:** Elizabeth Wambui, Calistus Wilunda, Hermann Pythagore Pierre Donfouet, Bonventure Mwangi, Taddese Alemu Zerfu, Tewoldeberha Daniel, Olivia Agutu, Betty Samburu, Daniel Kavoo, Lydia Karimurio, Pilar Charle Cuellar, Emily Keane, Lilly Schofield, James Njiru, Martin Chabi, Lucy Gathigi Maina, Peter Okoth, Judith Raburu, Grace Gichohi, Alex Mutua, Charles Matanda, Elizabeth Kimani-Murage

**Affiliations:** 1 African Population and Health Research Center, Nairobi, Kenya; 2 The World Bank Health Nutrition and Population Global Practice, Washington, DC, United States of America; 3 International Food Policy Research Institute, Addis Ababa, Ethiopia; 4 UNICEF Eastern and Southern Africa Regional Office, Nairobi, Kenya; 5 UNICEF Kenya County Office, Nairobi, Kenya; 6 Ministry of Health, Nairobi, Kenya; 7 Action Against Hunger, London, United Kingdom; 8 Save the Children UK, London, United Kingdom; 9 Save the Children International, Nairobi, Kenya; 10 World Health Organization, Kenya Country Office, Nairobi, Kenya; PLOS: Public Library of Science, UNITED STATES

## Abstract

Child undernutrition is a persistent challenge in arid and semi-arid areas due to low and erratic rainfall, recurrent droughts and food insecurity. In these settings, caregivers face several challenges in accessing health services for sick and/or malnourished children, including long distances to health facilities, harsh terrain, and lack of money to pay for transportation costs to the health facilities, leading to low service coverage and sub-optimal treatment outcomes. To address these challenges and optimize treatment outcomes, the World Health Organization recommends utilizing community health volunteers (CHVs) to manage acute malnutrition in the community. This study explored the perceptions of community members regarding acute malnutrition treatment by CHVs in Turkana and Isiolo counties in Kenya. The study utilized a cross-sectional study design and included a purposive sample of caregivers of children, CHVs, officers who trained and supervised CHVs and community leaders in the intervention area. Focus group discussions and key informant interviews were used to explore perceptions towards the management of acute malnutrition by CHVs. Generally, caregivers and CHVs perceived the intervention to be beneficial as it readily addressed acute malnutrition treatment needs in the community. The intervention was perceived to be acceptable, effective, and easily accessible. The community health structure provided a platform for commodity supply and management and CHV support supervision. This was a major enabler in implementing the intervention. The intervention faced operational and systemic challenges that should be considered before scale-up.

## Introduction

Acute malnutrition is still a public health concern in sub-Saharan Africa, with the prevalence being highest in East and West Africa [1]. Climate shocks and prolonged periods of drought resulting in inadequate food and pasture for animals in arid and semi-arid pastoralist communities are the main causes of malnutrition in Northern Kenya. This vast arid and semi-arid land is faced with a lack of infrastructural and economic development perpetuating the poverty levels and inability to access timely healthcare [2].

In Kenya, the treatment of acute malnutrition has been health-facility-based, with the identification of malnourished children left to health workers, with most of the screening happening during outreach services. Due to far distances to health facilities, poverty related-constraints and lack of knowledge, appropriate healthcare is often delayed or not adhered to, therefore affecting the recovery of children [3].

Community health volunteers (CHVs), also called community health workers, are community members selected by the communities they serve, trained for a short period to provide specific primary health care services in the communities where they come from and supported by the health system [4]. CHVs have the potential to supplement part of the formal health system and increase the access of community members to selected health services, as with Integrated Community Case Management (iCCM) [5]. As an equity-focused strategy that extends the reach of public health services, iCCM provides timely and effective treatment of malaria, pneumonia, and diarrhea among children below five years of age in areas with limited access to healthcare [6]. Over the last five years, iCCM has evolved at varying paces in different countries [7]. In most countries, it is typically delivered by CHVs at the community level assessing and delivering treatment for childhood pneumonia with antibiotics, diarrhea with zinc and oral rehydration salts (ORS), and malaria with artemisinin combination therapy (ACT) [6]. In some countries, the package also includes identification of malnourished children and follow-up at home (but not treatment). With an appropriate policy, CHVs can address malnutrition beyond screening and referral. Including treatment of acute malnutrition in the iCCM package has the potential to increase access to malnutrition treatment. However, there is little programmatic experience regarding how this would work for CHVs. Ethiopia and Rwanda, have implemented iCCM with the treatment of malnutrition as an integral component. Through iCCM, a program in Rwanda showed an increase in the number of children receiving treatment for diarrhea and pneumonia and a decrease in under-5 mortality by 38% [8]. In Ethiopia, health extension workers (HEWs) generally correctly managed children with uncomplicated malnutrition [9]. Further, a review of several studies shows that CHVs can identify and treat uncomplicated severe acute malnutrition (SAM), reduce defaulting to under 8% and achieve cure rates beyond the minimum standard [10].

In its latest update, WHO has provided guidelines for the identification and management of acute malnutrition by community health workers subject to adequate training [11]. In Kenya, Integrated Management of Acute Malnutrition (IMAM) was launched in 2009 with the role of CHVs delineated as screening and referral of malnutrition cases, community mobilization, and awareness raising in response to the persistent burden of malnutrition in arid and semi-arid areas [12]. In recent years, the iCCM landscape in Kenya has shown progress towards wider application and potential scale-up of the approach. The involvement of CHVs in iCCM in Bondo, Kenya, has shown a positive impact on the performance of iCCM, especially in community health care-seeking and in improving the competency and performance of CHVs on the same [13]. iCCM is implemented within the larger Ministry of Health community health services structures despite facing some challenges including capacity; retention; motivation and supervision of CHVs, commodity supply, and referral systems [13,14]. However, while evidence from elsewhere shows that well-trained, equipped and supervised CHVs can screen and treat children with malnutrition [15,16], concerns over how this affects CHVs’ workload, performance and commodity management (including transportation, storage, and accountability) surround the prospects of integrating acute malnutrition treatment within iCCM. Moreover, there is limited information on the challenges or facilitators of this intervention in the Kenyan context.

This study aimed to describe the experiences of community members and CHVs on integrating the treatment of acute malnutrition by CHVs into iCCM in Isiolo and Turkana counties, and explore the enablers and challenges of this intervention. The study was conducted to inform acute malnutrition management policy and strategy in Kenya.

## Methods

### Study design

This qualitative study was nested within a broader study on integrating the treatment of acute malnutrition by CHVs into iCCM. The qualitative study was cross-sectional among participants of the treatment arm of a cluster randomized controlled trial [17]. In the treatment group, 16 CHVs in Turkana and 17 CHVs in Isiolo were trained by implementing non-governmental organizations (NGOs) on how to use simplified tools and protocols to identify and treat eligible malnourished children (127 in Turkana and 107 in Isiolo) at home in addition to providing the usual iCCM package. In the control group, CHVs provided the usual iCCM package, which involves only screening and then referring malnourished children to health facilities. Details of this intervention are available in the published protocol [17]. Data were collected in Septemeber 2019 at the end of the intervention period using in-depth interviews (IDIs), focus group discussions (FGDs) and key informant interviews (KIIs). FGDs explored the general perceptions of community members and CHVs towards the intervention whereas IDIs and KIIs were used to gather in-depth information from CHVs and other stakeholders and to triangulate some of the information gathered through FGDs. We report this study according to the consolidated criteria for reporting qualitative research [18].

### Study settings

The study was conducted in two sub-Counties in Kenya, namely, Loima sub-County in Turkana County and Isiolo sub-County in Isiolo County. These areas are described in a published protocol [19]. These study sites were selected based on the following pre-set criteria: high prevalence of acute malnutrition, long distances from households to health facilities, presence of functional community health units (CHUs) with CHVs, existence of a supply chain system for ready-to-use therapeutic food (RUTF) and ready-to-use supplementary food (RUSF), and presence of an iCCM implementing NGOs as detailed in the published protocol [19].

Qualitative data were collected only in intervention CHUs in the study sub-counties.

### Participants and sampling

All the participants were purposively selected. CHVs were mobilized to participate in the study through the Community Health Assistants (CHA), who are health officers in charge of the CHVs. With information about our intended interview, the CHAs communicated with the CHVs participating ahead of the planned interviews. A similar approach was used to inform and mobilize caregivers on planned interviews through the CHAs and CHVs. In total, interviews were held with 99 participants from both Turkana and Isiolo. The study participants of FGDs were CHVs and caregivers from the units that participated in the intervention and were interviewed separately. Each FGD had 8–12 participants. These CHVs had been trained on how to screen, classify and treat acute malnutrition in the community and were therefore critical in providing information about their experience with the training and implementation of what they had been taught. Participants of KIIs were officers overseeing the implementation in each county. Some were overseeing CHVs, others were overseeing the supply of nutrition commodities, and others were overseeing the implementation of the program. Altogether, these officers were included to provide information on their experiences with the program implementation from their various capacities of oversight in the intervention. None of the individuals approached to participate in the study declined to participate or dropped out during the study.

### Data collection

Data were collected in September 2019, eight months after the start of the trial, by trained research assistants (RA) using pretested open-ended interview guides.

Data collection was done at the health facility in each CHU. The research team was composed of two RAs who were fluent in the local language and one research officer. One of the RAs interviewed participants while the other one took notes. The research officer supervised data collection but did not sit in the interviews. RAs regularly reported to the research officer and debriefed on each interview after it happened. Six FGDs with caregivers who had received and completed treatment from CHVs were done to understand their experiences and perceptions concerning the services. Two of these groups were composed of male caregivers and the other four groups with female caregivers. Information on their perceptions towards the intervention, the services they received and their level of satisfaction with CHV services, things that worked well through the intervention and those that did not work well. Four FGDs were carried out with CHVs to get their opinions and experiences on how they were trained, how they implemented what they had learnt and the intervention in general, challenges encountered and what worked well. We also interviewed CHAs to understand their opinions on supervising CHVs and commodity management. As this intervention was implemented by two organizations, one in Isiolo and the other in Turkana, program staff from both of these organizations were interviewed to capture their recommendations based on their experiences in implementing every aspect of the intervention For the same reason, interviews were also conducted with the Ministry of Health officers working closely with the implementing organizations in each sub-county.

The interviews were conducted in the local language or Kiswahili. FGDs lasted an hour on average while KIIs lasted for 40 minutes. While the lead interviewer prompted the question to the interview participants, the note-taker took down observations and emergent issues during the interview to capture the key responses provided by the participants. All interviews were audio-recorded and transcribed verbatim by bilingual (local language and English) speakers to ensure accuracy.

### Data analysis

The themes in this study were inductively drawn from the objectives of this study. The research team reviewed the transcripts to identify emerging themes and sub-themes that were used to develop a codebook. All transcripts were then coded along these themes and sub-themes using NVivo version 11 by one assigned coder. The coding was reviewed by the research team. Triangulation by comparing results from different groups of respondents and data collection methods was performed to check the consistency of the results. Quotations have been presented to speak to the themes and sub-themes. Participants have been anonymized to observe their confidentiality.

### Ethical considerations

The study protocol and tools were approved by the African Medical Research Foundation (AMREF) Health Africa Ethical and Scientific Review Committee (P416/2017). Written informed consent was obtained from all the interview participants. This process was carried out in the local language. Participants who could not write signed their consent using a thumbprint. The study was conducted following the principles of the Declaration of Helsinki and no incentives were offered for participation.

## Results

### Participants’ characteristics

Most interviewees were female caregivers who were mothers of malnourished children recruited in the intervention study and were married (Table 1). More than half of the FGD participants (47/86) had no education. Majority of the participants were self-employed and younger than 35 years.

**Table 1:** Characteristics of interviewees in Isiolo and Turkana.

	FGDs	KIIs
	(N = 86)	(N = 13)
**Sex**		
Male	24 (28%)	5(38%)
Female	62 (72%)	8 (62%)
**Age, years**	** **	** **
<20	5 (6%)	0
20–25	24 (28%)	0
26–30	17 (20%)	5 (38%)
31–35	15 (17%)	5 (38%)
>35	22 (26%)	3(24%)
**Marital status**	** **	** **
Not married	9 (10%)	0
Married	77 (90%)	13(100%)
**Education level**	** **	** **
No education	47 (55%)	0
Primary education	20 (23%)	0
Secondary education	19 (22%)	0
Tertiary education	0	13 (100%)
**Occupation**		
Unemployed	29 (34%)	0
Employed	0	13 (100%)
Self-employed	57 (66%)	0

This study exposed a range of issues regarding the treatment of malnutrition by CHVs. The results are summarized in Table 2 under three broad themes: 1) community perceptions towards CHVs screening, classifying, treating and following up acute malnutrition cases, 2) enablers of CHVs screening, classifying, treating and following up acute malnutrition cases and 3) challenges faced in implementing the intervention.

**Table 2:** Summary of themes and sub-themes.

**Community perceptions towards CHVs screening, classifying, treating and following up acute malnutrition cases**
Caregivers’ perceptions towards management of acute malnutrition by CHV CHVs’ perceptions of the intervention
**Enablers of CHVs screening, classifying, treating and following up acute malnutrition cases**
Sensitization of community members Supportive community health structures CHV proximity to caregivers Immediate deployment of CHVs after training
**Challenges faced in implementing the intervention**
Care-giver related challenges CHV related challenges Challenges with commodity supplies and stock-outs

### Community perceptions towards CHVs screening, classifying, treating and following up of acute malnutrition cases

#### Caregiver perceptions towards CHVs managing acute malnutrition

Overall, caregivers who received the services (treatment of children with acute malnutrition by CHVs in the community) were happy with the proximity and accessibility of the CHVs’ treatment services. They felt relieved because they did not require to walk long distances to obtain the treatment services. They were also pleased that CHVs were available to attend to them when health facilities were not open, for example over the weekends.

Community members perceived CHVs to be effective in treating malnutrition. They felt that the treatment provided by CHVs was similar to that provided in health facilities and were generally satisfied with the services they were receiving. Community leaders who were privy to the CHVs’ activities also expressed this sentiment.

*“I was very happy when the child was recruited to the program. The child improved weight, increasing in kilos. The child was dull before but after receiving the supplements child is now lively and very ok …”*

        FGD, Caregivers, Isiolo

*“…According to me, the perception of people in the village is that the work of CHV is good even when you compare the medicine they administer in the villages with the hospital one, the villagers say they are all okay, all is good because the drugs the CHVs administer to children while in the villages is that which they have got from the hospital meaning that that medicine is good…So generally the community is appreciating the work done by the CHVs…”*

        KII, Community leader, Turkana

When asked about the specific type of services they were receiving from the CHVs, caregivers mentioned treatment for malnutrition and education on the appropriate use of therapeutic feeds, and treatment for fever and malaria. Besides the treatment of children, caregivers reported having been counselled by CHVs. They mentioned receiving health education on malaria prevention, child care practices, breastfeeding, child feeding practices, food security, and hygiene and sanitation, as illustrated below.

*“…They [CHVs] have told us that before you give the baby anything, first breastfeed. Before you give him the plumpy nuts you breastfeed him… When the baby suckles, you give him water then after a short time, like 30 minutes you can give him food…”*

        FGD, Female Caregivers, Isiolo

*“…The most important thing the CHV instructed me to do is to make extra cleanliness around my locality, because if I do this, even the other diseases will not be closer to the child. Like making sure that the food is well covered from houseflies, the household utensils are clean and well kept away from houseflies, washing hand when you get out of the toilet and closing the toilet so that houseflies do not come out.…”*

        FGD, Female Caregivers, Turkana

#### CHVs’ perceptions of the intervention

CHVs, like caregivers, found the community management of acute malnutrition effective, accessible and convenient for the caregivers because it helped them to overcome the barrier of distance to the health facility. Further, because CHVs visited households and screened children, those with acute malnutrition were detected and treated earlier. They also felt the intervention equipped caregivers with knowledge to care for their malnourished children. The intervention also gave caregivers the confidence to seek treatment.

*“…Treating malnutrition at home was very good because there are many defaulters who would feel tired of going to hospital…Treating them at home has helped us because you know that mother, you go to her and tell her what to do…so you follow up on that.”*

        FGD, CHVs, Isiolo

*“…According to me, this one is better than the previous method, because the mothers of the sick children can now access the medicine in their villages without travelling a long distance. Now the doctors (CHVs)are the ones visiting them in their houses…”*

        FGD, CHVs, Turkana

CHVs also reported that their work brought with it an opportunity to learn new skills and practice them, hence becoming a source of motivation.

*“…To my side, I say the work has been good, we are learning more things from it, we are gaining experience as we work. We have been treating children and they are responding well, whoever will not respond we take him/her to the hospital…”*

        FGD, CHVs, Turkana

### Enablers to CHVs screening, classifying, treating and following up acute malnutrition cases

#### Sensitization of community members

Organizations implementing the intervention executed community sensitization activities as they commenced the implementation of the intervention. This was important as it not only informed community members but also built their trust in the CHVs to treat their children. Community members knew CHVs and their backgrounds and so it was necessary to inform and assure community members about the new role of CHVs. As a result, caregivers were largely welcoming of the CHVs’ services, as highlighted below.

*“…We did a lot before we started treatment because of course this was new to the community and building the confidence of the community members to help them know that the CHVs can now assess and treat…So building that confidence among the community members and telling them that these people are not quacks; they are people we have trained…”*

        KII, Implementing NGO staff, Isiolo

*“…after training we could not initially start off treatment because we had to do community engagement meetings with the community leaders and other people to inform them about the research and just to promote their participation in this process. We ensured that we had a start off meeting with the county management committee. So we ensured that we did that and we also held meetings. We had our own start off meetings so that we could share our work plans and tell them the objectives of the research.”*

        KII, implementing NGO staff, Turkana

#### A supportive community health structure

The availability of a supportive community health structure (CHS) enabled the provision of a framework for supervision, mentorship and commodity management. The intervention was implemented within CHUs that were already operational. In this structure, each CHV is assigned a designated households and is supervised by a CHA. Most CHVs were pleased with the consistent support supervision provided. They expressed satisfaction with the level of responsiveness that they received from their supervisors. This boosted their morale, confidence and competence. In both sub-counties, the CHA was the link to commodities.

*“…In my opinion it has been easy because we all know the day you give the child commodities. If you are giving tomorrow, the CHA sets her/his program and comes like today, everybody to have all her/his commodities so that when you go tomorrow you shouldn’t have stress of not giving those things. I think it has been very easy.”*

        FGD, CHV, Isiolo

*Provision of a stipend*. While in the CHVs in Turkana did not receive any cash stipend for their work during the study, those in Isiolo received a monthly cash stipend of Ksh 3000 (US$ ~30) from the program implementers. This motivated the CHVs in Isiolo because their work became an income source. On the other hand, CHVs in Turkana were very dissatisfied for not receiving any support as their county government had delayed to pay them an earlier promised stipend. There was a distinct difference in motivation between CHVs in Isiolo and those in Turkana. Unlike in Turkana, CHVs in Isiolo were more committed and consistent with treatment.

*“…I would also say that the little support that we give them, the incentives has really worked. It is one thing for one to close down their business and move to do household visits and sit down and start assessing a child and doing treatment …So that appreciation by the organizations that are supporting them has really worked towards motivating them and it is something I feel the county should pick up. You know telling them that please treat these children for us without anything is really demoralizing…”*

        KII, Implementing NGO Staff, Isiolo

*“Another challenge, me personally as a CHV, I visit my household, sometimes I see this work there is nothing it is helping me with. I am helping the community and yet me personally I am not benefiting, there is no motivation, sometimes you just…but you just decide let me just help my community.”*

        FGD, CHV, Turkana

*CHVs’ proximity to caregivers and close follow-up*. CHVs perceived that their close follow-up of caregivers increased compliance with good feeding and care practices thereby facilitating quicker recovery of the malnourished children.

*“What contributes to them getting well quickly is the follow-up by CHVs… for now they are followed up closely by the CHV that is what makes them to recover quickly…Also…there is that health talk, also you tell her that she maintains hygiene; wash her hands, wash the hands of the child and then give her/him those things not to give those other children or not to mix in food.”*

        FGD, CHVs, Isiolo

*“…There has been a difference. The children being managed at the community level are recovering faster. They are not staying long in the malnutrition program. Most are recovering very fast. Maybe it is because of close follow up. We are saying like for those at the health facility, they take long to recover because there is no follow-up …”*

        KII, CHA, Turkana

Because the CHVs were available to follow-up children they were treating, the treatment was regular and children rarely dropped out before recovery. Caregivers who found the distance to health facilities challenging were helped by the community-based treatment and did not default as they usually would. As the caregivers noticed the progress of their children, they were motivated to continue with treatment until full recovery.

*“…We used to refer people. The first day they go (to the hospital), the second day they don’t go. Defaulters have filled this register because they don’t go they say this side is far, this side there are many people, they say they don’t have time. Now we have made work easy for them…Even parents are pleased…Where she stays is where I bring her medicine. I measure her child. Even when I come, when I just enter her door you can see she is smiling. So you see even that mother is relaxed…”*

        CHVs, FGD, Isiolo

#### Immediate deployment of CHVs after training

Because CHVs went out and started applying their knowledge and skills immediately after being trained, they could easily rememberwhat they were taught and practice it correctly. Over time, both CHAs who were supervising CHVs and program implementers felt that CHVs had progressively learnt how to screen children well using the mid-upper arm circumference (MUAC) tape, and distinguish and treat accordingly the type of malnutrition presented. CHVs were also perceived to be managing well diarrhea and fever.

*“…In my case, I am seeing there is improvement in terms of screening and maybe also in terms of referrals for the cases they cannot manage from the community to the facility. There is a good linkage between the community and the facility, so I have seen they are also managing well in terms of commodities and therefore the cases they can actually manage within the community…”*

        KII, SCHMT, Turkana

*“…Where they have been treating very well, it is on the malnutrition cases. The malnutrition cases and also, on diarrhea cases, and also the fever of the children…Yes, they know how to manage a small child.”*

        KII, CHA, Isiolo

### Challenges experienced by CHVs in screening, classifying, treating and following-up children with acute malnutrition

#### Caregiver-related challenges

*Lack of caregiver confidence in CHVs’ capacity to treat children*. At the onset, a few caregivers were doubtful of the CHVs’ ability to treat their children. They questioned the skills and competencies of CHVs to attend to their children, with some remaining adamant until they began to witness other children getting better. A few refused to have their children treated by CHVs and only changed their perception after being adequately informed about the intervention. Some caregivers refused CHVs’ treatment due to perceived adverse effects of the supplementary and therapeutic food and previous misinformation. Culture also seemed to play a role. In both Isiolo and Turkana, Young CHVs were despised by older caregivers because of their age.

*“…In the very beginning I had doubts as to what authority they had and where they got the ability to do that work because most of the time there are questions that may arise later, say when that child doesn’t heal well and I usually hear of cases where you can treat a child and maybe you don’t have that education or skills. Therefore, my fear was if for sure they have the ability, they have the experience and they have the authority to do what they are doing…”*

        FGD, Male Caregivers, Isiolo

*“…In my view, what has made their work to be hard first, you know majority of the CHVs in my area, most of them are young people. So because they are youths and community members are older and according to tradition, when a younger person speaks in front of older people, even if she/he is talking about important or wise things, most of the times what they are saying is taken lightly…”*

        KII, Community Leader, Turkana

*Unmet treatment expectations by community members*. The community lacked understanding about the CHV’s scope of work. Some community members continued to expect and request CHVs to treat children older than five years. This was also observed among caregivers with children experiencing conditions that were beyond the scope of the CHV’s work. CHVs often had to clarify their roles and sometimes this put them in conflict with community members who could not understand this.

It was common for CHVs to receive requests to treat other ineligible community members such as elderly persons and children older than 5 years.

*“They have been told it is children from a certain age to a certain age and it becomes a gap to those who don’t get that service which we would also like to get from them…There was even a time I experienced a mother bringing a child and the child is given those services and then you hear the mother saying ‘I also have this and this problem’ but she doesn’t have that other equipment to help the mother…”* FGD, Male caregivers, Isiolo

*“…The other thing is what CHV said about old people, whenever they get sick they come to us asking for medicine and when you say you are dealing with children alone it becomes segregation…”*

        FGD, CHV Turkana

*Caregiver refusal to be referred to the health facility*. When a CHV referred a child to the health facility for reasons such as non-response to treatment, the presence of comorbidities, or lack of treatment commodities, some caregivers resisted the referral or refused to comply. Caregivers refused referral mainly because of the existing barriers to health facility service utilization, such as perceived staff attitude and long distance to health facilities.

*“…I had one child who had severe acute malnutrition, so I followed up the child but the weight went down, the child was not moving at all, was still stuck down there and the weight continued to reduce. So I sent them to the facility. At the facility also, they followed up the child. I heard they were told to go to general… the big hospital. So recently, because she was referred to me by another CHV, I told that CHV to make sure the child has gone even if it is transport they lack, we see how we will make sure she/he has gone. And I hear even now the child is not breastfeeding … So all that time I have started to tell her to take to the sister [faith-based facility health worker] she says she cannot go to the sister because she will start telling her ‘…Why can’t you hold the baby well and what…’”*

        FGD, CHVs, Isiolo

*“…One of the challenges is when a mother refuses to be transferred. You see you get a very malnourished child …And a CHV … refers the child…a malnourished child who has regressed in MUAC… So, the mother now refuses, ‘I am not going anywhere!’ So, that is one challenge. There is that refusal of mothers to be transferred. Because when these services came and they are close, they really appreciated but again when they are referred to the facility they feel that distance …”*

        KII, Nursing officer, Turkana

*Caregiver absenteeism during CHV follow-ups*. Some CHVs found it difficult to follow-up some caregivers because the caregivers did not keep appointments, causing delays and uncertain waiting time. In other instances, caregivers would be unavailable for follow-up as per the treatment schedule. Often this absenteeism was due to caregivers prioritizing other issues such as seeking a livelihood for the family or migrating in search of animal pasture. Related to this was the lack of male involvement in caring for children. If a CHV visited a household and found a male caregiver, this visit was ineffective because the male caregiver would not provide the required information because he does not attend to the child. CHVs were more assured dealing with female caregivers because they were directly involved in childcare and hence more informed about the child’s condition.

*“…You see here in our area, you know the father says a child belongs to the mother, even when you find him with the child there he cannot bother until the mother comes…I wait for the mother to come, it is the mother who knows the issues of this child…”*

        FGD, CHVs, Isiolo

*“…The challenges that I would say is, first, availability; that this CHV can go to a certain household to treat a child, she/he finds the parent is not there, she/he has gone with the child somewhere. So this CHV misses somebody to treat. She/he comes back without treating that person…”*

        KII, Community Leader, Turkana

*“…Sometimes you meet these people they are pastoralists. They move with their animals, so you meet them the way in a village, they normally make it a temporary village. So when they see that there is no pasture there for the animals, they move to another place. So, it is very hard also for us to trace those mothers…”*

        KII, CHA, Isiolo

#### CHV-related challenges

*Inadequate number of CHVs working in vast areas*. Both community members and CHVs said that CHVs were too few to cover the target area and population. This inadequacy was compounded by the vast distance between households. CHVs visited households mainly by walking and reaching community members was difficult.

*“They [CHVs] are very few, so they cannot attend to all those people. For instance, 10 people have come at the same time. So one person cannot attend to that village at once, it’s just that one person is assigned to one village…There is a big area here, like Marerei and other areas like Chokaa, Nasuroi, Mawe area, even up to behind there…So there, the CHV will not reach unless he/she has a motorbike or goes and spends the night there…”*

        FGD, Male Caregivers, Isiolo

“*Another challenge is the distance where you are going to check that child…*. *So you have to plan yourself so that you are able to reach that child*. *And reaching that child*, *the day you are going maybe you have gotten a casual job somewhere else*, *you know that is not the only job you are doing*, *you have to fend for your children*. *So the challenge also is the issue of transportation*.*”*

        FGD, CHVs, Isiolo

*Low numeracy and literacy skills among some CHVs*. Successful training and implementation of the intervention relied heavily on CHVs’ education level. During the training, CHVs with low literacy and numeracy levels required more teaching time to comprehend the content and master the skills. During the implementation, these CHVs needed help to fill their registers and reports. They often delayed to submit their reports because they relied on the availability of literate persons to help withdocumentation.

*“…If you look at the levels of education for CHVs, you will see it varies; there are those who are really educated up to form four [final year of secondary education], there are those that reached class seven, class four, even to those that just know how to write. …you know if you are not educated, you will not perform at the same level with those that are educated… There are also those who understand but they do not know how to write. They go and assess a child they know that the child is on red but for filling the sick child recording form, it becomes a challenge…”*

        KII, SCHMT, Isiolo

*“…There are documentation challenges especially with those that are not able to read and write. Sometimes the ones that help them to document might not be available and hence we cannot get their reports.”*

        KII, CHA, Turkana

*Technical challenges faced by CHVs in treating children*. As CHVs progressed in the treatment of children, some tasks remained difficult to execute for some of them despite their training and practice. Issues specific to iCCM such as the use of rapid diagnostic kits for malaria, filling the Sick Child Recording Form and conducting clinical assessments for respiratory tract infections (e.g. checking for chest in-drawing and counting the respiratory rate) were difficult for someof CHVs. A few CHV had difficulties in taking the MUAC measurement, weighing and recording weight measurements and filling treatment registers. These challenges were seen in both Isiolo and Turkana especially among CHVs with low literacy.

*“…There is a challenge when it comes to this area of severe pneumonia… If the child is sleeping, they do not know if it’s a normal sleep or abnormal sleep, so they could get challenges on that…As I said we have a few CHVs that have low understanding capacities, but when we went on-job training, they now get and understand what we are doing. For the MUAC taking, there are CHVs who do not know how to use these MUAC tapes properly.”*

        KII, CHA, Isiolo

*“…In terms of treatment…I think some CHVs are not even able to use the Rapid diagnostic test kits and all that…also, I realized in terms of the amount of rations for the therapeutic feeds what they are supposed to be given to this child…there is still a bit of problems and all that and even filling up of those registers. So that is a few specific CHVs but not all…If we can pull up in terms of refresher and OJTs [on-the-job training], they can improve…”*

        KII, SCHMT, Turkana

“…*When we are talking about pneumonia, we are talking about a child who has a danger sign of chest in-drawing…to observe or to identify what this child is undergoing, maybe is suffering from pneumonia, that you find there is a challenge. We talk about fast breathing, those ones we are using the beads, we are using the timer for them to count those you find that some of them are not counting properly*. *It needs someone like me to be near and tell them to do like this, do like this during the supervision…”*

        KII, CHA, Turkana

*Conflict between volunteerism and working to earn a livingfor CHVs*. CHVs are unpaid volunteers who also need to fend for themselves and their families. In trying to strike a balance between serving the community and bread winning, some CHVs prioritized the latter, failing to execute their tasks as required. This was more common in Turkanawhere CHVs did not receive a stipend.

In terms of managing the work versus managing personal schedules, some CHVs mentioned that they separated the days given to volunteer work and personal work while others mentioned that they run both tasks concurrently. All CHVs unanimously agreed that scheduling of the tasks was dependent on them and they would normally schedule their activities conveniently.

*CHVs’ increased workload*. As CHVs began treating children in the community, they spent more time than usual in households with sick children. Depending on the illness, the CHV would be required to make return visits until the child recovers or is referred to the health facility. Because this was not the foreseen mode of operation, the CHV’s workload increased. When asked about how they viewed their current workload given the need to treat children in the community, most CHVs felt the workload was high and in some cases, overwhelming at the onset. As the intervention progressed, some CHVs felt that the workload decreased as children in the community recovered and remained well.

*“…I visit them in a month, once or if there is one whose child is malnourished, I have to visit her/him according to the treatment I am giving her/him, if it’s MAM or SAM. If she/he has malaria I have to go visit her/him after 3 days, I have to attend to her/him…”*

        FGD, CHVs, Isiolo

*“…So there was that concern about workload but we had to*, *actually just bargain with them that*, *because he was telling me it is making them not to do the other roles*, *to look for what to eat*, *to fend for their families*. *So*, *that was their concern that this has brought increased roles…I think when they manage the cases*, *malnutrition cases dropped and they realized that the cases are dropping*, *they are able to manage until now like him*, *like this week we met*, *he doesn’t have cases*. *He has discharged all the children but he said he thought it was a difficult thing*.*”*

KII, Program Officer, TurkanaCHVs experienced a challenge in managing their workload. While some were able to visit all their assigned households within a month, many could not achieve this. This was because some CHVs had too many households depending on the population of their villages. Further, these households were sparsely distributed and the distances involved made it difficult to visit their assigned households at the required frequency.

*“…You know there is this MOH 514 [reporting form]*, *it requires that in one month you should be done with all your households*. *You see now*, *you should be done with your households even if they are 40*, *there are those who have 50*, *60*, *70 and you will ensure that you finish these 70 by the last date of the month…*”

FGD, CHVs, Isiolo

*“…Now, they [CHVs] plan their schedules on a weekly basis. A CHV will plan the households that they can visit in a week. The next week they go to other households. However, there are those CHVs that have a lot of households like 150 households. Sometimes it is hard to cover all those households, but they plan on how to visit all those households…”*

        KII, CHA, Turkana

*Lack of a stipend for the CHVs*. There was a universal perception that the provision of a stipend would motivate CHVs to do their work. While in Isiolo the implementing organization provided a small stipend to the CHVs, the same was not done for CHVs in Turkana where demotivation due to the lack of a stipend emerged strongly. This problem was aggravated by the need of CHVs to volunteer and still provide for their families.

*“Now that we are not paid and the households are many, you find sometimes you cannot make it to reach all of them because you have to go and look for food for yourself. … we request to be supported with something small for motivation…”*

        FGD, CHVs, Turkana

#### Challenges with commodity supplies and stock-outs

Commodity distribution was initially difficult and time-consuming in Isiolo. CHAs were required to physically confirm the presence of a sick child before releasing commodities to the CHVs. This process was slow and demanding for CHVs and CHAs and resulted in treatment delays.

After the commencement of the intervention, both counties experienced an interruption of commodity supply, which in turn interrupted CHVs’ treatment plans. However, the situation was resolved by restocking commodities and reassuring CHVs.

When the supply of commodities was interrupted,CHVs were inconvenienced by the needto make multiple trips to health facilities to collect commodities. Also, when CHVs did not place orders in good time, the facility request form did not capture the true needs of the community unit. This resulted in an inadequate commodity order and supply through the supply chain up to the final recipient.

*“…The organization, especially on the side of malnutrition has not been covered well because you go and find a child, and after getting the child has malnutrition that is when you will start making calls to be looked for those supplements. And then the child has done let’s say you have found her/him on yellow, you have given treatment for 2 weeks…. So two weeks again when they are over, the date she is supposed to come for them, that date again you have to look for that day or even when you look for them you are told maybe they have not reached or and maybe let’s say is on Friday you are told until Monday, Saturday and Sunday there is no work…”*

        FGD, CHVs, Isiolo

*“…When I have not received those commodities myself, or they have been delivered and they are finished…it is two or three weeks before they are delivered again…So, the number of commodities I have seen being delivered is also small since they do not even last for two months.”*

        KII, Nutritionist, Turkana

## Discussion

This study has explored community perceptions towards integrating the management of acute malnutrition into iCCM in Isiolo and Turkana counties and the enablers and challenges of this integration. Overall, CHV management of acute malnutrition was received well in the community and its implementation was deemed effective. The community health structure was essential in the training, supervision and on-the-job training for CHVs in the delivery of this service. However, community management of malnutrition experienced challenges including the administration of commodities, vast distances and transport barriers and demotivation of CHVs due to lack of a stipend to compensating for their time and effort.

The study found that the intervention was generally acceptable by the community members. This study was conducted in low-income arid areas where residents are overly dependent on pastoralism and are faced with a severe household food insecurity and poor dietary intake. Because of long distances between households and health facilities, caregivers struggle to access health services for their children. Because the treatment of malnutrition requires a consistent review of sick children, the need to visit health facilities recurrently for caregivers with a sick child becomes daunting given other household demands they face. This intervention brought malnutrition treatment services closer to households thereby eliminating several barriers caregivers were facing in seeking care at health facilities. CHVs were pleased to be involved in this intervention that conveniently filled a long-standing health service delivery gap in the community. The close proximity between CHVs and caregivers facilitated easy access to malnutrition screening and treatment, health education, follow-up of malnourished children and caregiver compliance with treatment in general. An increased demand of CHV services including what was not in their scope is an indication of the acceptability of the services by caregivers. This finding is similar to that from a study in Mumbai India, where caregivers were found to be receptive of the home visits and growth monitoring by frontline health workers terming the visits as encouraging and beneficial [19]. Studies undertaken in various contexts also show that the regular screening of children in the community and timely response with treatment by CHVs can resolve uncomplicated malnutrition [10].

The study found several enabling factors for the treatment of malnutrition by CHV. The Community Health Strategy [20], an already existing structure, provided a supportive framework for operational organization, supervision and mentorship, commodity management and reporting. Support supervision and mentorship by the CHAs boosted the confidence of CHVs in to implementing their tasks. CHVs gradually improved their performance based on the regular feedback they received from their supervisors. The CHAs also provided the necessary support on operational issues such as accounting for commodities. A similar support structure was reported in Bangladesh where the decentralized nature of community health workers backed by good supervisory support and backstopping was successful and contributed to the recovery of children in the program [15]. In this study, as in others [21], sensitization of the community was crucial and served to address doubt and provide clear expectations among community members while fostering their confidence in the services provided by the CHVs. It also promoted community participation and ownership of the intervention.

This study found that the implementation of management of acute malnutrition by CHVs had challenges. Operationally, there was an inadequate number of CHVs to deliver the services effectively lack of stipends for the CHVs and interrupted supply of commodities and drugs. At an individual level, CHVs had varied mastery of skills with education levels having a bearing in their ability to learn, master, deliver and report their service provision. While there was an effort to improve their skills through supervision and on-the-job training, low education level was a barrier to their uptake of skills and effective service delivery Operational challenges in the supply of commodities to CHVs has also been reported in Ethiopia [22] alongside the need for CHVs to adequately master treatment skills. In Odisha India, community workers implementing a similar intervention experienced increased workload, limitations in technical know-how and relatable challenges in the flow of commodities [21]. In this Kenyan study, some challenges were resolved over time as the intervention progressed while other challenges like commodity flows, recruitment and remuneration of CHVs required the intervention of policy makers, donors and implementing partners.

Commitment from the policy makers, donors, implementing partners and the communities is fundamental for CHVs to deliver health services to communities [19]. There is a need to address issues such as the age and education levels of CHVs, core competencies and remuneration. As already seen in the case of iCCM [23], there is no doubt that CHVs can generate greater demand for and access to health services. This can only be sustained if CHVs are trained, motivated, and supervised to work within a supportive environment that guarantees sufficient backstopping and operational efficiencies [24]. Equally important is the need to sensitize and inform community members for their buy-in and support of this intervention.

## Strengths and limitations

The strengths of this study include a collection of data from different categories of participants yielding a wide range of perceptions. This provides for wide representative viewpoints. Triangulation of the results from different categories of respondents and data collection methods increases the validity and reliability of our findings. Nonetheless, one potential limitation is that this study was conducted in the setting of a field trial that lasted for a short period and it is uncertain how the experiences would change with long-term implementation in a real-world setting. Secondly, the study supported the implementation of iCCM and the management of acute malnutrition at the same time. Even though most CHVs had prior training with iCCM, this was the most consistent, prolonged implementation of it alongside the management of malnutrition.

## Conclusions

It is clear from the findings that the management of acute malnutrition in the community by CHVs was well received as it met the treatment needs of the community and addressed barriers that previously hindered successful treatment. The intervention motivated CHVs by providing a means to solve long-standing community challenges they had previously faced with malnourished children and equipping them withknowledge and skills in acute malnutrition management.

The challenges experienced were similar in both counties and should be taken as learnings that can inform the implementation of similar community-based interventions. As this study was implemented over six months, the intervention’s barriers and challenges should be viewed in terms of necessary measures required at the health system level.

Interventions structured around CHVs should take into account the entire community health structure as a fundamental resource for the successful operation of community interventions. Resourcing this structure adequately extends a stable and reliable health in the community.
